# Study on the Polycrystalline Mechanism of Polycrystalline Diamond Synthesized from Graphite by Direct Detonation Method

**DOI:** 10.3390/ma15124154

**Published:** 2022-06-11

**Authors:** Shi-Yuan Shang, Yi Tong, Zhi-Chao Wang, Feng-Lei Huang

**Affiliations:** State Key Laboratory of Explosion Science and Technology, Beijing Institute of Technology, Beijing 100081, China; bit0211001@163.com (S.-Y.S.); tongyi@bit.edu.cn (Y.T.); 3220190154@bit.edu.cn (Z.-C.W.)

**Keywords:** graphite, polycrystalline diamond, multinuclear growth, inert additive, grain size

## Abstract

In this paper, a polycrystalline diamond was synthesized by the direct detonation method using graphite as the carbon source. By comparing the numbers of the obtained diamond particles and the original graphite particles, it was found that when the graphite phase transformed into the polycrystalline diamond during the detonation process, a single graphite particle would form multiple diamond nuclei, and the nuclei would grow simultaneously to form polycrystals. Accordingly, a validation experiment was designed, which added different ratios of inert additives while keeping the ratio of graphite to hexogen (RDX) unchanged. It was found that increasing the ratio of inert additives within a certain range could increase the grain size of a polycrystalline diamond, which is consistent with the obtained polycrystalline mechanism.

## 1. Introduction

The direct detonation method is a method of synthesizing a diamond by mixing carbon sources, such as graphite and carbon black, with high-energy explosives. It uses the high temperature and high-pressure environment formed by the energy released from the explosive detonation to transform the carbon source into a diamond. The diamond synthesized by graphite and RDX is a polycrystalline diamond. A polycrystalline diamond is a polymer formed by the coalescence of multiple single crystals and has good wear resistance, thermal stability, impact toughness, and isotropic properties. The research on polycrystalline diamonds started from a kind of “carbonado” natural diamond, which is a polycrystal formed by the polycrystallization of many diamond crystals and some other impurities. In addition, natural polycrystalline diamonds include “ballas” and “framesite”. The artificial synthesis methods of polycrystalline diamonds include the detonation synthesis method [1,2,3], vapor deposition method [4,5,6], static high-temperature and high-pressure method [7,8,9], sintering method [10,11], etc. Regarding the study of the polycrystalline mechanism of detonation synthesis of polycrystalline diamonds, people mainly focus on the mechanism of phase transformation from graphite to diamond. Some scholars [12,13] believe that the graphite phase that transforms to diamond is a diffusion phase transition, while some scholars [14,15,16,17] believe that it is a martensite phase transition. In addition, some others [18,19,20] believe that both mechanisms exist.

Kleiman et al. [12] use a unique flash-heating hemispherical implosion system to generate high temperature and pressure pulses which are applied to graphite/metal mixtures. After recovery of the mixtures, transmission electron microscopy (TEM) and x-ray and electron diffraction are adopted to analyze them. The results show the existence of diamond, different forms of graphite, and carbynes in the samples. After in-depth study and discussion, a formation mechanism of a diamond is proposed that relies on a solid-vapor-liquid-solid (SVLS) sequence of phase transformations. Zhang W.J. [13] compares the martensitic transformation mechanism and thermal diffusion mechanism of the shock-induced phase transition of graphite to diamond, and analyses the shock wave propagation characteristics in loose media. It is concluded that the effect of temperature is much greater than pressure and shear stress, which supports the graphite phase transformation into diamond as a thermal diffusion mechanism.

Hirai et al. [21] adopted a rapid-cooling technique to quench the graphite sheets shock-compressed to 65 GPa and 3700 K to obtain two modified forms of carbon. An n-diamond was obtained from the fastest cooling rates, which is very similar to the structure of a cubic diamond. The other form, which was obtained from the relatively slow cooling rate, is similar to the i-carbon structure prepared by the ion-beam technique. The n-diamond is a metastable form, similar to a hexagonal diamond, which is converted from graphite through a martensitic transformation. Mundy et al. [22] used ab initio molecular dynamics to simulate the phase transformation of graphite to diamond under shock compression in conjunction with a multi-scale shock technique (MSST). The simulations showed a novel short-lived layered diamond intermediate is formed within a few hundred femtoseconds upon shock loading at a shock velocity of 12 km/s (longitudinal stress > 130 GPa). Then, the cubic diamond was formed. The transition was martensitic. With the help of large-scale molecular dynamics simulations, Zhu et al. [23] reported a mechanism whereby the diamond nuclei in the graphite matrix propagate in two preferred directions, among which the graphite [120] is about 2.5 times faster than [001]. Consequently, cubic diamond is the kinetically favorable product, while only a few hexagonal diamonds can exist as the twins of cubic diamonds.

Yamada et al. [18] studied the shock-induced phase transition of ordered pyrolytic graphite to a diamond-like phase, it was observed that the lowest transition onset pressure was 19.6 GPa and it was considered that the phase transition, in that case, was martensitic. However, if there are voids between particles in the ordered pyrolytic graphite, diamond-like carbon and diamond will be obtained when the graphite is loaded at a pressure up to 15 GPa. In that case, it was considered that the phase transition was caused by the release of distortional energy stored in graphite particles, that is, the diffusional-controlled reconstructive mechanism. Sumiya et al. [19] characterized the ultra-hard polycrystalline diamond synthesized from graphite by direct conversion under static high pressure by transmission electron microscopy and electron diffraction. The results showed that a polycrystalline diamond had a mixed texture of a homogeneous fine structure and a lamellar structure. The experimental results suggest that diamond particles in the homogeneous fine structure were transformed from graphite in the diffusion process, while diamond layers in the lamellar structure were formed in the martensitic process from graphite via the hexagonal diamond phase. Zhuk et al. [24] examined the microstructure of graphite quasimonocrystal recovered after dynamic loading to a pressure of 35–45 GPa. Only small amounts of cubic diamonds and recrystallized graphite were detected. The relaxation time of the transformation (~10 ns) and the degree of the transformation (~70–80 vol.%) were determined by means of measurements of the electrical resistivity during the loading up to 26 GPa. They proposed that two simultaneous processes take place at pressures higher than 20 GPa: (i) relatively slow diffusive graphite to diamond transformation localizes a defect structure in the zones and (ii) highly oriented graphite transforms to a diamond-like phase with a density of about 3.2 g/cm^3^ at zero pressure. This transformation has fast, martensitic kinetics and is reversible.

The formation mechanism of polycrystals in diamonds has been little studied. Cao Y. et al. [25] believe that after the small particles of the graphite phase transform into diamond during the detonation process, they will combine to form large polycrystalline diamond particles because of the large surface area and surface activity. However, the large particles of graphite are directly formed into large polycrystalline diamond particles.

In this paper, the number of graphite particles is estimated by estimating the size of graphite, and then the number of polycrystalline diamond particles is calculated. In comparing them, the polycrystalline mechanism of a polycrystalline diamond is obtained. At the same time, inert additive experiments were designed to verify the proposed polycrystalline mechanism.

## 2. Particles Number Comparison Experiment

The 400-mesh size graphite was selected as the external carbon source and RDX was used as the high energy explosive. The experiment was conducted by the direct detonation method. The graphite mass fraction was 16%, the charge density was 1.5 g/cm^3^, and the experimental conditions are shown in Table 1.

The size of 400 mesh graphite particles is 38 μm, and the graphite is approximated as a spherical particle with a diameter of 38μm for calculation, then the volume of one graphite particle is 2.299 × 10^−13^ m^3^. The density of graphite is between 2.09–2.33 g/cm^3^, and taking 2.09 g/cm^3^ for calculation, then the mass of a single graphite particle is 4.8 × 10^−7^ g. Therefore, 112.9 g of graphite contains 2.21 × 10^8^ graphite particles.

**Figure 1:**
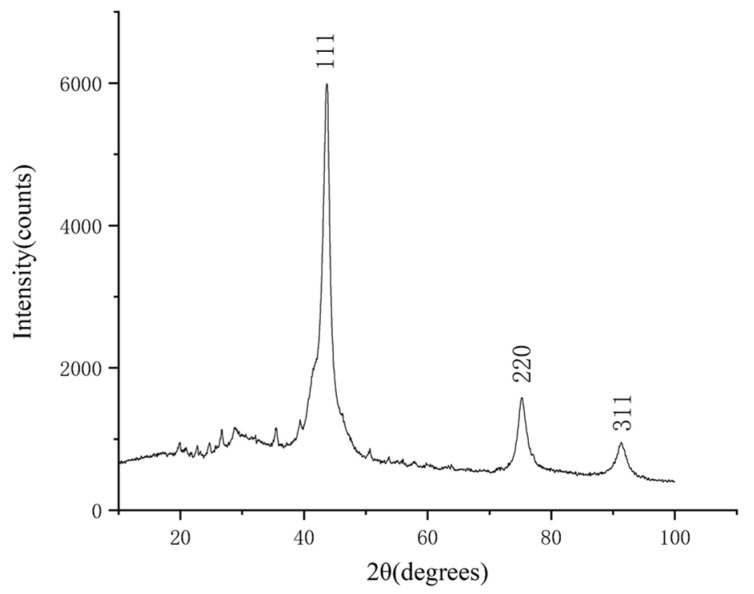
The XRD pattern of the purified product. The horizontal coordinate indicates the diffraction angle and the vertical coordinate indicates the intensity of the diffraction peaks. The crystal planes corresponding to the three diffraction peaks are indicated.

After purification of the detonation soot, 29.8 g of the purified product was obtained. The X-ray diffraction (XRD) characterization of the purified product was carried out by a Bruker D8 ADVANCE diffractometer, and the test conditions were as follows: fitting limits are 10° to 100°, and the step size is 0.02°. The result is shown in Figure 1. The XRD pattern shows three distinct diffraction peaks near 43.7°, 75.2°, and 91.3°, which correspond to the (111), (220), and (311) crystal planes of a cubic diamond by comparison with the standard card (JCP: 03-065-6329). The heights of other diffraction peaks are obviously lower than these three diffraction peaks, and the number of other diffraction peaks is very small, which indicates that the main component of the product is a cubic diamond with few impurities. The analysis of the diffraction data shows that the full width at half maximum (FWHM) of the diffraction peak corresponding to the (111) crystal plane is 3.82° and the grain size of the diamond is 21.3 Å, which is obtained by the Scherrer equation.

**Figure 2:**
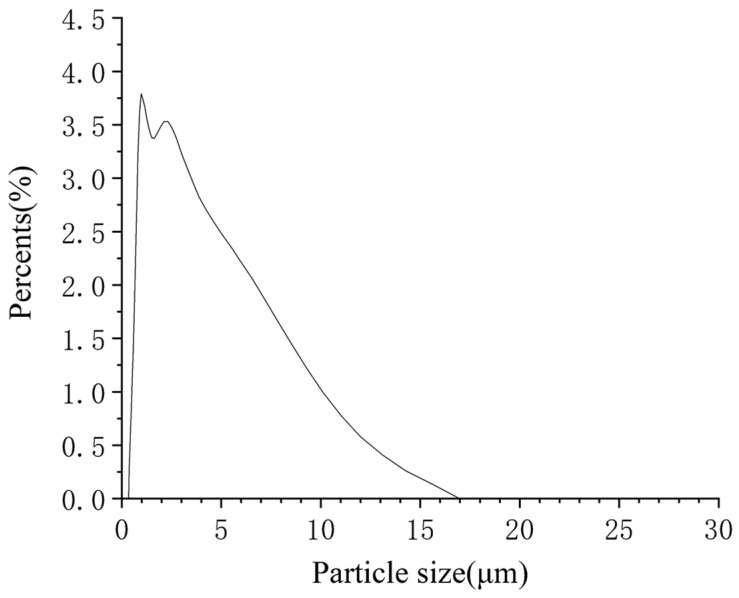
Laser particle size distribution of the purified product. The horizontal coordinate indicates the particle size and the vertical coordinate indicates the percentage of particles in the total sample at the corresponding size.

The laser particle size analysis of diamond products was carried out by Horiba LA-920 particle size analyzers and the particle size distribution is shown in Figure 2. It can be seen from the figure that the size of diamond particles is distributed in the range of 0.3–16 μm. An in-depth analysis of the data shows that the average particle size by particle number (D_MN_) is 0.704 μm, which means this diameter is equal to the average of the diameters of all particles. The average particle size by volume (D_MV_) is 2.826 μm, which means the volume of one particle with this diameter is equal to the average of the volumes of all particles. The D_MN_ is smaller than the D_MV_, which indicates that there are more small particle diamonds. The volume of a diamond calculated by D_MV_ is 1.18 × 10^−17^ m^3^ and the density of a diamond is 3.5 g/cm^3^, then the mass of a single diamond particle is 4.13 × 10^−11^ g. Therefore, a 29.8 g diamond has 7.22 × 10^11^ particles. The number of diamond particles is obviously more than the number of graphite particles which indicates that, in the process of conversion of graphite to diamond, a single graphite particle commonly forms multiple diamond particles.

**Figure 3:**
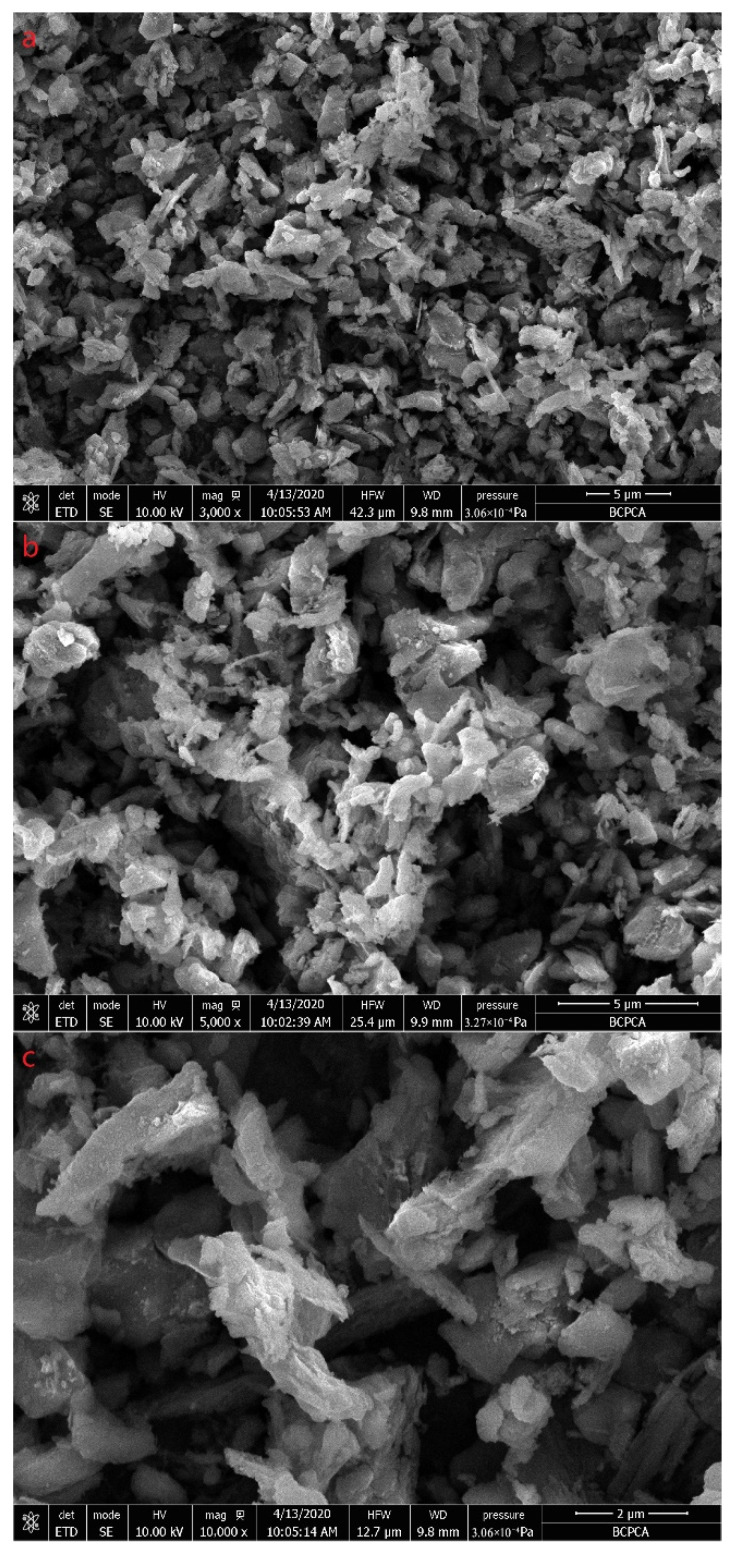
SEM images of the purification products. The magnification of (**a**) is 3000, the magnification of (**b**) is 5000, and the magnification of (**c**) is 10,000.

Figure 3 shows SEM images of the purification products, which were tested by the FEI Tecnai G^2^ F30 Field Emission Gun Transmission Electron Microscope with SE mode. Most of the diamond particles are less than 5 μm in size as seen in image a, which is consistent with the results of the laser particle size analysis. It can be seen from the figure that the morphology of diamond particles is various, such as blocky, lamellar, columnar, etc. As shown in image c, some diamond particles have lamellar structures on the surface. These lamellar structures are similar to the morphology of graphite.

When the temperature and pressure formed by the explosion are in the diamond phase region of the carbon phase diagram, the carbon atoms in a single graphite particle will form multiple diamond nuclei, which will grow in all directions to form grains simultaneously. Grain boundaries will be formed when adjacent grains come in contact and growth will stop in that direction. These adjacent grains will coalesce to form polycrystals. If all carbon atoms of the graphite particle are not completely transformed into diamond, then a hybrid structure will be formed which is a mixture of a polycrystalline diamond structure and graphite structure. This mixed structure of diamond and graphite may or may not break during the subsequent expansion phase of the detonation product due to collisions with other particles, but the graphite in this mixed structure will be removed during the purification process. Thus, multiple polycrystalline diamond particles are obtained. If there happens to be graphite surrounded by diamond grains, then the graphite will be protected from oxidation removal by the diamond. If all carbon atoms of graphite particles are converted into diamond, lamellar diamond particles with the same morphology as graphite will be obtained. In addition, the polycrystalline diamonds transformed by adjacent graphite particles will also bond to each other to form larger polycrystalline diamond particles under suitable pressure and temperature.

## 3. Verification Experiment of Polycrystalline Mechanism

Under this polycrystalline mechanism, the more diamond nuclei formed for one graphite particle, the smaller the average grain size of the diamond will be when the graphite particle is completely transformed into a diamond. Combining with the nucleation rate in the solid-state phase transition theory of metals [26]

(1) $$I=n\nu exp\left(-\frac{Q+W}{kT}\right)$$

where *I* is the nucleation rate, *n* is the number of atoms per unit volume of the parent phase, *ν* is the frequency of the atomic vibrations, *Q* is the atomic diffusion activation energy, *W* is the nucleation energy for the formation of a critical nucleus, *k* is Boltzmann constant, *T* is phase transition temperature.

(2) $$W=\frac{16\pi \sigma^{3}}{3{\left(\Delta G_{V}-\varepsilon \right)}^{2}}\;,$$

where *σ* is the interface energy per unit area between the new phase and parent phase, *ε* is the new phase elastic strain energy per unit volume, and Δ*G_V_* is the free energy difference per unit volume between the new phase and the parent phase. The bigger the difference between the theoretical and actual phase transition temperature of the new phase and the parent phase, the bigger the Δ*G_V_* is, and the smaller the *W* is.

The increase in temperature can increase the nucleation rate, for as the temperature increases, i.e., *T* becomes larger, *W* becomes smaller, and I eventually becomes larger.

When the temperature reaches the theoretical phase transition temperature of graphite to diamond, i.e., the higher the temperature is, the more diamond nuclei will be formed, and the average grain size of the diamond will be smaller. Therefore, an experiment was designed for verification.

Under the condition of graphite and RDX ratio of 84:16, the detonation synthesis experiment was carried out by changing the content of inert additives, including polypropylene (PP), polyethylene (PE), and melamine formaldehyde resin (MF). The specific experimental scheme and results are shown in Table 2.

It can be seen from the above table that the mass of detonation soot is much higher than the mass of added graphite but is close to the sum of graphite and inert additive mass, which indicates that the inert additive does not participate in the chemical reaction during the detonation process. If the inert additive participates in chemical reactions during the detonation process, the mass of the inert additive will reduce, and the mass of the detonation soot will not be close to the sum of the mass of graphite and inert additive. It is assumed that in the process of detonation, PP, PE, and MF do not participate in the detonation reaction, and no other chemical reaction occurs. Under this assumption, the Urizar formula, empirical formula, and Custor method were used for the theoretical calculation of the explosive charge. The results are shown in Table 3.

It can be seen from the calculation results, that the group with low inert additive content has significantly higher detonation pressure and detonation temperature. There are two reasons, one is the higher content of explosives and the other is the higher density of the charge.

After detonation, soot was obtained, and chemical purification was carried out to remove the amorphous carbon, graphite, inert additives, and other impurities to obtain a pure diamond. The purification results are shown in Table 4.

**Figure 4:**
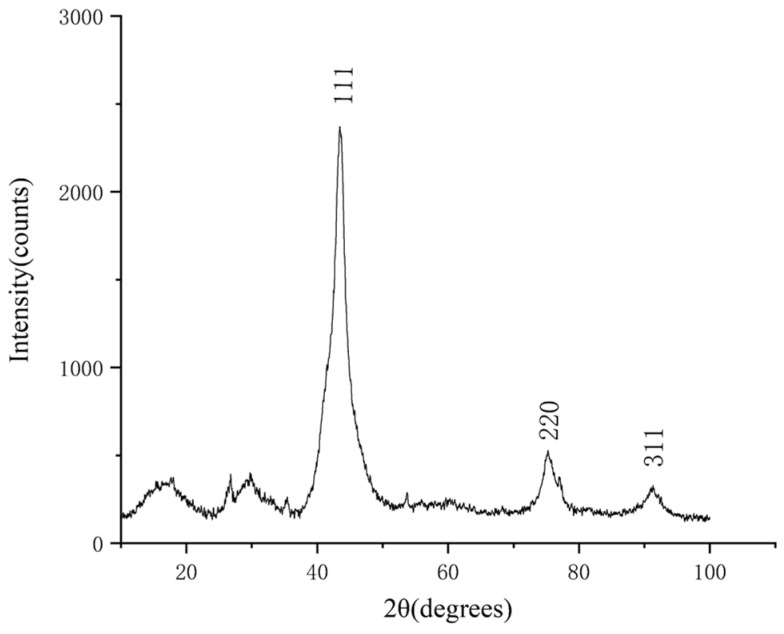
The XRD pattern of the purified product of the 10% PP experimental group. The horizontal coordinate indicates the diffraction angle and the vertical coordinate indicates the intensity of the diffraction peaks. The crystal planes corresponding to the three diffraction peaks are indicated.

**Figure 5:**
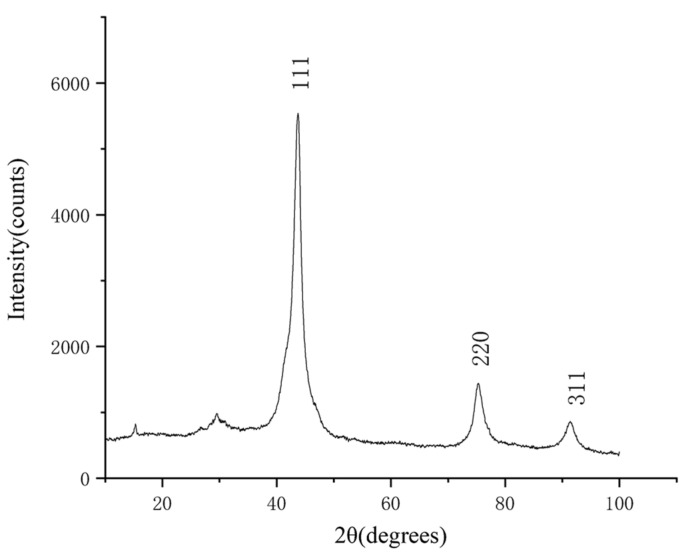
The XRD pattern of the purified product of the 20% PP experimental group. The horizontal coordinate indicates the diffraction angle and the vertical coordinate indicates the intensity of the diffraction peaks. The crystal planes corresponding to the three diffraction peaks are indicated.

**Figure 6:**
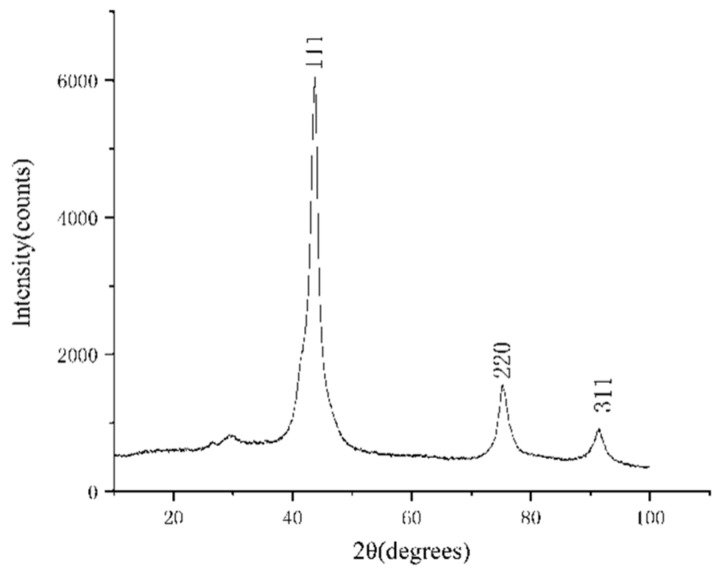
The XRD pattern of the purified product of the 10% PE experimental group. The horizontal coordinate indicates the diffraction angle and the vertical coordinate indicates the intensity of the diffraction peaks. The crystal planes corresponding to the three diffraction peaks are indicated.

**Figure 7:**
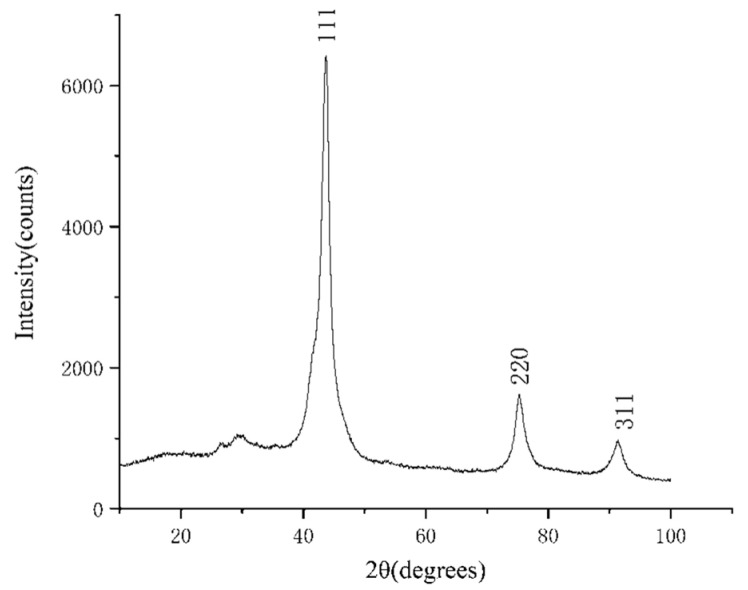
The XRD pattern of the purified product of the 20% PE experimental group. The horizontal coordinate indicates the diffraction angle and the vertical coordinate indicates the intensity of the diffraction peaks. The crystal planes corresponding to the three diffraction peaks are indicated.

**Figure 8:**
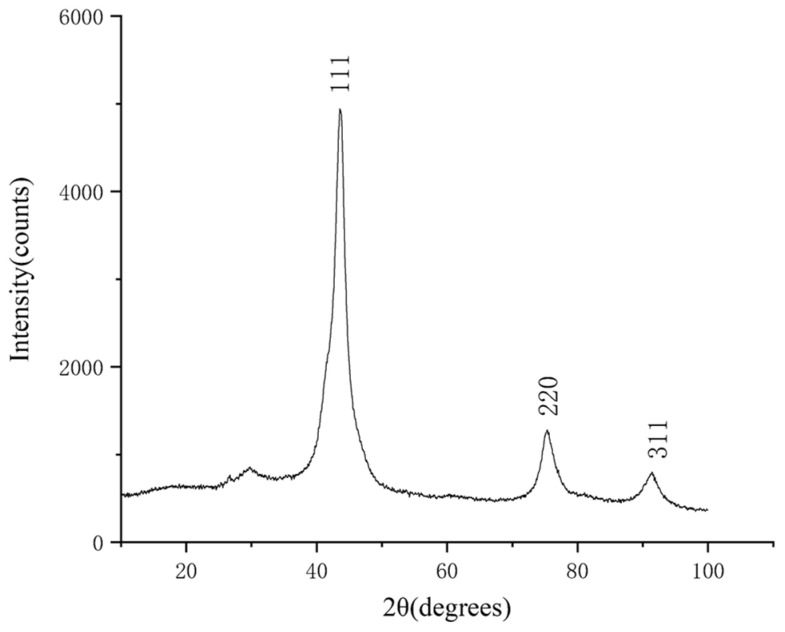
The XRD pattern of the purified product of the 10% MF experimental group. The horizontal coordinate indicates the diffraction angle and the vertical coordinate indicates the intensity of the diffraction peaks. The crystal planes corresponding to the three diffraction peaks are indicated.

**Figure 9:**
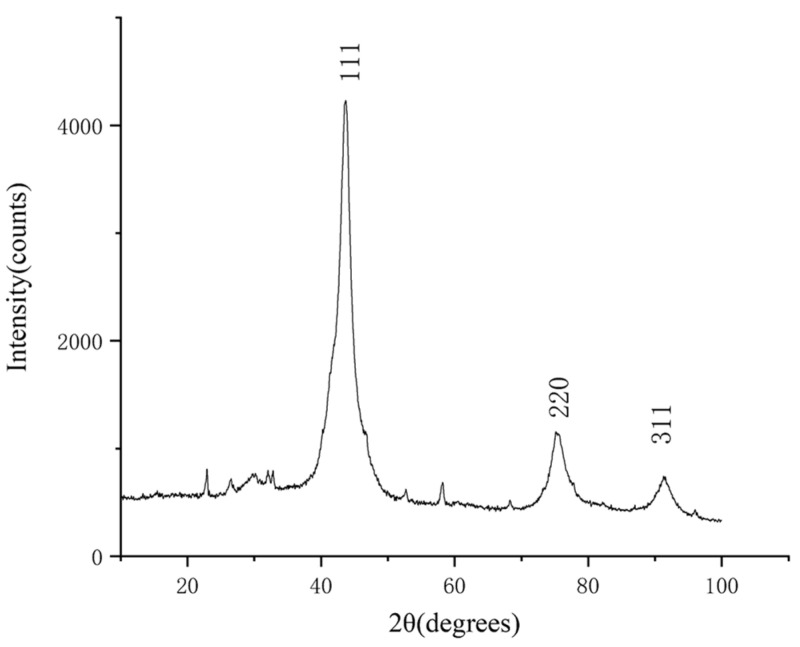
The XRD pattern of the purified product of the 20% MF experimental group. The horizontal coordinate indicates the diffraction angle and the vertical coordinate indicates the intensity of the diffraction peaks. The crystal planes corresponding to the three diffraction peaks are indicated.

**Figure 10:**
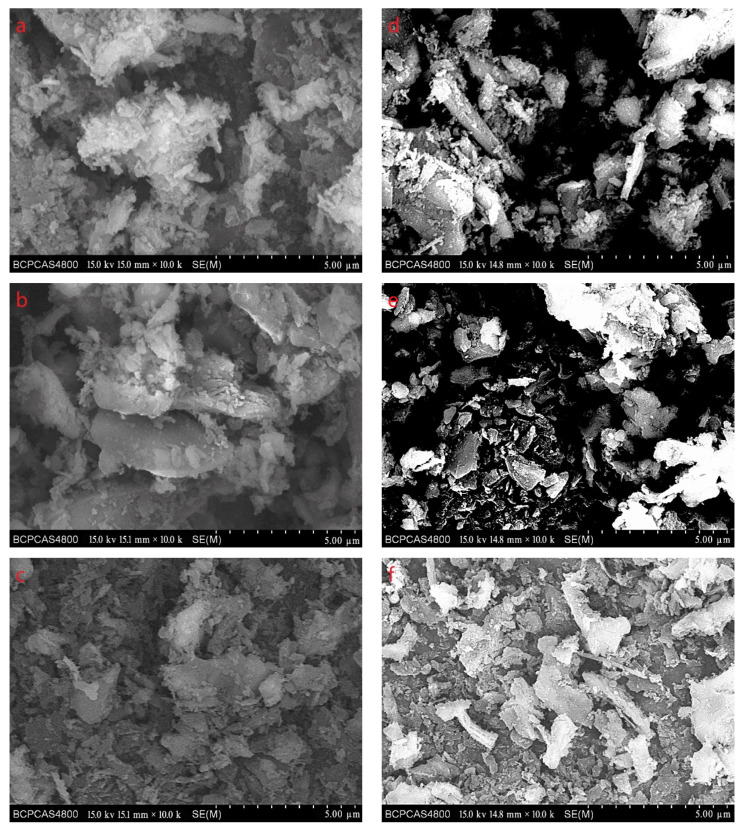
The SEM images of the purified products of the verification experiment. Images (**a**–**f**) are SEM images of 10% PP, 10% PE, 10% MF, 20% PP, 20% PE, and 20% MF experimental groups, respectively.

The purified products were characterized by XRD and SEM. The SEM was tested by a Hitachi S-4800 high resolution scanning electron microscope, and the acceleration voltage was 15 kv. The results are shown in Figure 4, Figure 5, Figure 6, Figure 7, Figure 8, Figure 9 and Figure 10.

According to the XRD patterns, there are three diffraction peaks near 43.7°, 75.3°, and 91.3° in each of the above XRD patterns. Compared with the standard card (JCP:03-065-6329), it was found that these three diffraction peaks correspond to the crystal planes of (111), (220), and (311) of a cubic diamond, which indicates that the purified product is diamond. In addition, fewer miscellaneous peaks indicate that the product is purified. Figure 10 shows the SEM images of the purified products of the verification experiment. It can be seen from Figure 10 that the diamond morphology of the verification experiments is similar to the diamond morphology obtained from the particle number comparison experiment which contains various morphologies such as blocky, columnar, and lamellar. The diffraction data are analyzed to obtain the FWHM of the diffraction peak corresponding to the (111) crystal plane and the grain size of the corresponding product. The results are shown in Table 5.

It can be seen from the above table that the diamond grain size of the group with lower inert additive content is also lower. This is consistent with the inference based on the proposed polycrystalline mechanism. In other words, when the content of inert additives is lower, the detonation temperature and detonation pressure of the mixed charge are higher. In that case, the nucleation rate of the graphite phase transformation into diamond will be higher, and graphite will form more diamond cores. Finally, the average grain size of the diamond obtained is smaller.

## 4. Conclusions

In conclusion, during the detonation process, the graphite will form multiple diamond cores and grow simultaneously to form diamond grains. In this process, there are two mechanisms for the formation of polycrystals. Firstly, diamond grains in one graphite particle will stop growing and form grain boundaries when they come in contact with each other, and a type of polycrystal will be obtained. The second is due to temperature, pressure, and size effects, the first type of polycrystals formed in adjacent graphite particles are bonded with each other to form a second type of polycrystal.

Because the number and position of the diamond grains composing the first type of polycrystals are different, the shape of the first type of polycrystals will be different. The number, shape, and position of the first type polycrystals composing the second type polycrystals are different, so the shape of the second type of polycrystals will be different. This eventually leads to polycrystalline diamonds synthesized by detonation in various shapes such as blocky, columnar, and lamellar.

In addition, under this theory of polycrystalline mechanism, when the added graphite can be fully transformed into diamond, the higher the content of inert additives is, the lower the detonation temperature and pressure of the charge are, and the fewer diamond nuclei formed on the same graphite particle. Eventually, the size of the diamond grain will be larger. The experimental results also prove this inference.

## Figures and Tables

**Table 1:** Particle number comparison experimental scheme and results.

Diameter of Charge (mm)	Density of Charge (g/cm^3^)	Mass of Graphite (g)	Mass of RDX (g)	Mass of Detonation Soot (g)	Mass of Purified Product (g)
50	1.5	112.9	592.73	139.4	29.8

**Table 2:** Experimental verification scheme and results.

Inert Additives	Diameter of Charge (mm)	Density of Charge (g/cm^3^)	Mass of Graphite (g)	Mass of RDX (g)	Mass of Detonation Soot (g)	Ratio of Detonation Soot to Graphite	Ratio of Detonation Soot to Charge
10% PP	30	1.4	8.64	45.36	16.86	195.14%	28.10%
20% PP	30	1.24	7.68	40.32	14.46	188.28%	24.10%
10% PE	30	1.56	8.64	45.36	16.63	192.48%	27.72%
20% PE	30	1.42	7.68	40.32	19.26	250.78%	32.10%
10% MF	30	1.67	8.64	45.36	13.42	155.32%	22.37%
20% MF	30	1.59	7.68	40.32	13.82	179.95%	23.03%

**Table 3:** Calculation results of detonation parameters of the charge.

Inert Additives	Density of Charge (g/cm^3^)	Detonation Velocity—D (m/s)	Detonation Pressure—P (PGa)	Detonation Heat—Q (J)	Detonation Temperature—T (℃)
10% PP	1.4	6826	16.77	4903	3390
20% PP	1.24	6295	12.84	4767	3296
10% PE	1.56	7345	20.58	5038	3405
20% PE	1.42	6872	16.47	4920	3321
10% MF	1.67	7607	22.74	5132	3399
20% MF	1.59	7240	19.30	5064	3341

**Table 4:** Purification results from detonation soot.

Inert Additives	Mass of Purified Sample (g)	Mass of Purified Product (g)	Purification Yield	Ratio of Purified Product to Charge	Ratio of Purified Product to the Carbon Source
10% PP	10.004	0.076	0.76%	0.21%	1.48%
20% PP	10.061	0.282	2.80%	0.67%	5.27%
10% PE	10.021	0.445	4.44%	1.23%	8.54%
20% PE	10	0.255	2.55%	0.82%	6.40%
10% MF	9.787	0.513	5.24%	1.17%	8.14%
20% MF	10.003	0.420	4.20%	0.97%	7.55%

**Table 5:** The XRD data of the purified products.

Inert Additives	Density of Charge (g/cm^3^)	FWHM (°)	Grain Size (Å)
10% PP	1.4	4.86	16.7
20% PP	1.24	2.5	32.5
10% PE	1.56	2.84	28.6
20% PE	1.42	1.93	42.2
10% MF	1.67	3.75	21.7
20% MF	1.59	2.51	32.4
